# Complete Genome Sequence of Border Disease Virus Genotype 3 Strain Gifhorn

**DOI:** 10.1128/genomeA.01142-13

**Published:** 2014-01-16

**Authors:** Ulrik Fahnøe, Dirk Höper, Horst Schirrmeier, Martin Beer, Thomas Bruun Rasmussen

**Affiliations:** DTU National Veterinary Institute, Technical University of Denmark, Lindholm, Kalvehave, Denmarka; Institute of Diagnostic Virology, Friedrich-Loeffler-Institut, Greifswald-Insel Riems, Germanyb

## Abstract

The complete genome sequence of the genotype 3 border disease virus strain Gifhorn has been determined; this strain was originally isolated from pigs. This represents the consensus sequence for the virus used to produce the bacterial artificial chromosome (BAC) cDNA clone pBeloGif3, which yields a virus that is severely attenuated in cell culture.

## GENOME ANNOUNCEMENT

Border disease virus (BDV) belongs to the genus *Pestivirus*, which includes other important animal pathogens, such as bovine viral diarrhea virus (BVDV) and classical swine fever virus (CSFV). BDV causes disease mainly in small ruminants, but cattle, pigs, and other wildlife may also be infected; indeed, the BDV genotype 3 (BDV-3) strain Gifhorn was originally isolated from both infected pigs and sheep kept on the same farm (1, 2). The BDV genome consists of a positive-sense RNA approximately 12.3 kb in length, which encodes a single polyprotein that is posttranslationally cleaved to form structural and nonstructural proteins. The genetic diversity of BDV is high, with multiple major genotypes reported (3). Complete genomic sequences have been described for genotypes BDV-1 (strains BD31 [4] and X818 [5]), BDV-2 (strain Reindeer-1 [6]), and BDV-4 (strain H2121 [3]). In addition, recently, the complete genome of BDV JSLS12-01, which is closely related to that of BDV-3 Gifhorn, has been reported (7). A complete sequence for BDV-3 Gifhorn was published previously, but this was derived from a bacterial artificial chromosome (BAC) cDNA clone, pBeloGif3 (GenBank accession no. GQ902940); virus rescued from this cDNA displays severe growth attenuation in cell culture (8). The complete genome sequence of the BDV-3 Gifhorn isolate has now been determined.

Viral RNA was extracted from BDV-3 Gifhorn-infected sheep fetal thymoid (SFT-R) cells, and full-length viral cDNAs were amplified by long reverse transcription-PCR (RT-PCR) as previously described (8). cDNA was prepared from viral RNA according to the manufacturer’s protocol (Roche, Mannheim, Germany; cDNA Rapid Library Preparation Materials and Methods Manual). Sequencing libraries were generated from cDNA or from RT-PCR products using the SPRIworks Fragment Library System II (Beckman Coulter, Krefeld, Germany) and were sequenced using a 454 FLX (Roche). Newbler (Roche) was used for *de novo* assembly and for mapping of the reads using pBeloGif3 (GenBank accession no. GQ902940) as a reference sequence. Finally, the consensus sequences were aligned using MAFFT in the Geneious software platform (Biomatters).

The consensus sequence for the BDV-3 Gifhorn genome was obtained by replicate sequencing of RT-PCR products and RNA. The replicate samples generated the same consensus sequence, which was 12,325 nucleotides (nt) long. The polyprotein-coding sequence is 11,694 nt long and contains 3,898 codons. The 5′ and 3′ untranslated regions (UTRs) are 375 and 256 nt long, respectively. A comparison with the cloned Gifhorn genome sequence (from pBeloGif3, 12,326 nt) revealed a 1-nt deletion in the 5′ UTR and 11 nt differences in the coding sequence, including 8 that are nonsynonymous. The complete consensus sequence of this BDV-3 virus and its comparison with the pBelGif3 sequence should enhance our understanding of the factors that are important for viral attenuation. The generation of additional genomic data for BDV will assist further investigations into the properties of this group of viruses.

### Nucleotide sequence accession number.

The genome sequence of BDV-3 Gifhorn has been deposited in GenBank under the accession no. KF925348.
